# Interplay between the microalgae *Micrasterias radians* and its symbiont *Dyadobacter* sp. HH091

**DOI:** 10.3389/fmicb.2022.1006609

**Published:** 2022-10-13

**Authors:** Yekaterina Astafyeva, Marno Gurschke, Wolfgang R. Streit, Ines Krohn

**Affiliations:** Department of Microbiology and Biotechnology, Biocenter Klein Flottbek, University of Hamburg, Hamburg, Germany

**Keywords:** *Dyadobacter* sp. HH091, *Micrasterias radians*, microalgaebacteria interaction, synthetic early plant-bacteria system, symbiotic relations

## Abstract

Based on previous research, related to detailed insight into mutualistic collaboration of microalga and its microbiome, we established an artificial plant-bacteria system of the microalga *Micrasterias radians* MZCH 672 and the bacterial isolate *Dyadobacter* sp. HH091. The bacteria, affiliated with the phylum Bacteroidota, strongly stimulated growth of the microalga when it was added to axenic algal cultures. For further advances, we studied the isolate HH091 and its interaction with the microalga *M. radians* using transcriptome and extensive genome analyses. The genome of HH091 contains predicted polysaccharide utilizing gene clusters co-working with the type IX secretion system (T9SS) and conceivably involved in the algae-bacteria liaison. Here, we focus on characterizing the mechanism of T9SS, implementing the attachment and invasion of microalga by *Dyadobacter* sp. HH091. Omics analysis exposed T9SS genes: *gldK*, *gldL*, *gldM*, *gldN*, *sprA*, *sprE*, *sprF*, *sprT*, *porU* and *porV*. Besides, *gld* genes not considered as the T9SS components but required for gliding motility and protein secretion (*gldA*, *gldB*, *gldD*, *gldF*, *gldG*, *gldH*, *gldI*, *gldJ)*, were also identified at this analysis. A first model of T9SS apparatus of *Dyadobacter* was proposed in a course of this research. Using the combination of fluorescence labeling of *Dyadobacter* sp. HH091, we examined the bacterial colonisation and penetration into the cell wall of the algal host *M. radians* MZCH 672.

## Introduction

Algae and bacteria synergistically collaborate with each other, influence ecosystems, and represent various modes of interactions between organisms (Ramanan et al., 2016). The positive effect of bacteria on algal growth in the field of biotechnology, has changed the main concept of a mere contamination of algal cultures, considering bacteria as an important driver in this interaction (Lee et al., 2015; Shen and Benner, 2018). Strong associations between microalgae and bacteria have resulted in the evolution of a complex network of these cross-kingdom interactions and narrow specialization of different organisms (Krohn et al., 2013; Krohn-Molt et al., 2017; Cirri and Pohnert, 2019; Astafyeva et al., 2022).

Nowadays, it is recognized that the potential of the interactions between microalgae and microorganisms, determined by special applicability in aquaculture, aims to improve algal biomass production and to enrich this biomass with compounds of biotechnological interest such as lipids, carbohydrates, and pigments. The algal microenvironment may be altered by bacteria in ways that stimulate algal functions. The general bacterial attributes that may profit the interaction with microalgae, and which might affect their growth and photosynthetic activity, include adhesion, clumping factor, motility, chemotaxis, different secretion systems, quorum sensing and quenching systems, and synthesis of growth promoters (Luo and Moran, 2014; Brameyer et al., 2015; Shen and Benner, 2018; Astafyeva et al., 2022).

Previous research of microalgae-and photobioreactors-associated biofilm bacteria, identified that the majority of the observed microorganisms were affiliated with α-Proteobacteriota, β-Proteobacteriota, and Bacteroidota (Mouget et al., 1995; Davies et al., 1998; Krohn et al., 2013; Whitman et al., 2018). Further investigations have characterized the biotic interaction of microalgae and bacteria using metagenomic, transcriptomic, and proteomic approaches. In this research the microbiomes of microalga have been sequenced, and various bacterial strains affiliated with the algae have been isolated to answer, if the associated microbiota is specific for the microalgae and which role individual bacterial taxa play (Krohn-Molt et al., 2017). Thereby it was observed that effector molecules known from plant–microbe interactions as inducers for the innate immunity are already of relevance at this evolutionary early plant-microbiome level. Key genes involved in plant–microbe interactions were mostly affiliated with different mechanisms, including vitamin biosynthesis, transport and secretion systems, signal transduction, carbohydrate and lipid modification. The metatranscriptome analysis indicated that the transcriptionally most active bacteria, with respect to key genes commonly involved in plant–microbe interactions, in the microbiome of the *Chlorella* (Trebouxiophyceae), *Scenedesmus* (Chlorophyceae) and *Micrasterias* (Zygnematophyceae) belong to the phylum of the α-Proteobacteriota and Bacteroidota (Krohn-Molt et al., 2017).

Recent studies unveiled tight associations of microalga *Scenedesmus quadricauda* and bacteria using metatranscriptomic analysis, including physiological investigations, microscopy observations, photosynthetic activity measurements and flow cytometry. The crucial key features of overall plant-bacteria interaction covered different mechanisms with the involvement of transport and secretion systems (e.g., T6SS, T9SS), quorum quenching proteins (QQ), leucine-rich repeat proteins and enzymes (LRR) related to bacterial reactive oxygen species (ROS) tolerance, as well as the biosynthesis of vitamins (B_1_, B_2_, B_5_, B_6_ B_7_, B_9_ and B_12_). The metatranscriptome analysis demonstrated that within the microbiota of *S. quadricauda* the dominant species were affiliated with the genera of *Variovorax*, *Porphyrobacter* and *Dyadobacter*. Experimental and transcriptome-based evidences implied that within this multispecies interaction *Dyadobacter* was a key to alga growth and fitness, and is highly adopted to live in the phycosphere (Astafyeva et al., 2022).

Within this framework, we addressed the following questions in the current study. Which role do secretion systems play in these remarkable interactions? Is a direct cell-to-cell contact between the interaction partners required and what influence does bacterial QS have? To answer these questions, we used fluorescence labeling of bacteria and 4′-6-diamidino-2-phenylindole (DAPI) staining with confocal microscopy to determine the physical association of microalga cells with the *Dyadobacter* isolate HH091. Further, to get a deeper insight in this fascinating synthetic bacteria-microalgae model system, we have characterized the interactions of the isolate *Dyadobacter* sp. HH091 (Astafyeva et al., 2022), with the microalga *M. radians* MZCH 672 using transcriptome and genome analyses. These data expand our understanding of species-species interactions and identify several genes involved in the molecular basis of bacteria-alga interactions that can serve as an established synthetic plant-bacteria system. Therefore, the genome and metabolic potential of the bacterium *Dyadobacter* sp. HH091 is of particular interest in understanding bacteria-algae interactions.

## Materials and methods

### Microorganisms used in this study and cultivation media

*Micrasterias radians* MZCH 672 was obtained from the Microalgae and Zygnematophyceae Collection Hamburg (*MZCH*) and cultivated in WHM medium (Stein, 1973) at 20 ± 1°C and 100 ± 10 μmol photons m^−2^ s^−1^ with a 14/10-h light/dark period. To maintain the axenity of the algal culture, *M. radians* was treated with the antibiotic cocktail: penicillin G, streptomycin sulfate and gentamycin sulfate (100/25/25 mg/l) (Droop, 1967; Andersen, 2005; Lee et al., 2015; Astafyeva et al., 2022).

*Dyadobacter* sp. HH091 was isolated previously from a laboratory culture of *S. quadricauda* MZCH 10104 (Krohn-Molt et al., 2017; Astafyeva et al. 2022). The isolate was routinely grown in 5 ml of tryptone yeast extract salts (TYES) broth (Reasoner and Geldreich, 1985; Holt, 1993), at 22°C for 3–4 days at 200 rpm.

### Analysis of the flexirubin pigments in *Dyadobacter* sp. HH091

We experimentally validate the production of flexirubin by *Dyadobacter* sp. HH091 by exposing them to 50 μl 10 M KOH, which resulted in a change from yellow to orange/red if flexirubin pigments were present, followed by a neutralization step with 42 μl 12 M HCl, which resulted in a return to yellow pigmentation.

### Co-culturing procedure and conditions

*Micrasterias radians* MZCH 672 and *Dyadobacter* sp. HH091 were co-cultured in WHM medium at 20 ± 1°C and 100 ± 10 μmol photons m^−2^ s^−1^ with a 14/10 h light/dark period over a time period of 12 days. Therefore, 1 ml of *M. radians* was treated with an antibiotic cocktail of penicillin G, streptomycin sulfate and gentamycin sulfate in 50 ml of WHM medium to remove all bacteria. The antibiotic treatment was performed for 1 day. Afterwards, the microalga was centrifuged (5,000 rpm, 10 min) and washed two times with 1 ml WHM medium and finally resuspended in 50 ml of medium, where it was grown for 20 days. At the start of the experiment, each flask contained 50 ml of WHM, *M. radians* (OD_750nm_ = 0.007) and *Dyadobacter* sp. (OD_600nm_ = 0.05).

### *Dyadobacter* sp. HH091 transformation

The strain HH091 was transformed with modified plasmid pBBR1MCS-5-eGFP by electroporation according to standard methods, which resulted in bright green fluorescent colonies as observed by fluorescence microscopy (Sambrook and Russell, 2001). The plasmid contains the broad-host-range vector pBBR1MCS-5, providing a gentamycin resistance and the expression of GFP. Gentamycin was applied at 100 μg/ml, and the bacteria were grown as described previously (Droop, 1967; Andersen, 2005; Lee et al., 2015; Astafyeva et al., 2022).

### Confocal laser scanning microscopy

*Dyadobacter* sp. HH091 expressing eGFP was co-cultured with *M. radians* MZCH 672 and studied using a confocal laser scanning microscope (CLSM) Axio Observer.Z1/7 LSM 800 (Carl Zeiss Microscopy GmbH, Jena, Germany), which also included Z-Stack microscope techniques. The analysis of the CLSM images were done with ZEN software (version 2.3; Carl Zeiss Microscopy GmbH). DAPI staining procedure was used in microscopy investigations as described previously (Astafyeva et al., 2022). Modifications included the treatment with TrueVIEW Autofluorescence Quenching Kit (Vector Labs, SP-8400), which was employed to enhance staining and to lower the autofluorescence of chlorophyll of the microalga. Background autofluorescence occurring in the 600–700 nm range, makes it impossible to detect the bacteria transformed with plasmids expressing fluorescent proteins. The TrueVIEW Quencher is an aqueous solution of a hydrophilic molecule, which binds to chlorophyll electrostatically and lowers the fluorescence (Karpishin, 2018).

### Bacterial RNA isolation and sequencing

*Dyadobacter* sp. HH091 cells, separated by dialysing bags (Roth, Germany), were co-cultured with microalga for 1 week. Then bacterial cells were subsequently harvested, treated with RNAlater (Sigma, Germany) and frozen at −80°C. The samples were processed by Eurofins (Constance, Germany), where the RNA was isolated and assessed for QC. The RNA Integrity Number (RIN) for all samples was ≥8. Strand-specific cDNA library preparation from polyA enriched RNA (150 bp mean read length) and RNA sequencing was performed using the genome sequencer Illumina HiSeq technology in NovaSeq 6000 S4 PE150 XP sequencing mode. For further analysis fastq-files were provided.

### Bacterial RNA data analysis

RNA-seq analysis was performed using PATRIC, the Pathosystems Resource Integration Center.^1^ Trim Galore 0.6.5dev was used to remove adapters (Phred quality score below 20) (Krueger, 2012). RNA-Seq data was processed by the tuxedo strategy (Trapnell et al., 2012). All genes were selected with|log2 (fold change)| ≥ 1,5. The differentially expressed genes (DEGs) dataset was collected and used for further analysis. The volcano plot of the distribution of all DEGs was generated using A Shiny app ggVolcanoR (Mullan et al., 2021).

Carbohydrate-active enzymes were screened through local Blastp search in the database of carbohydrate-active enzymes (CAZymes).^2^ The database compiles categories of enzymes that act on carbohydrates, e.g., glycoside hydro-lases (GHs), polysaccharide lyases (PLs), glycosyltransferases (GTs) (Levasseur et al., 2013). Domain guided annotation based on conserved domains in *Dyadobacter* sp. HH091 was performed within the STRING database (Szklarczyk et al., 2021).

### Sequences obtained and GenBank submissions

RNA sequences obtained for this study were submitted to the European Nucleotide Archive (ENA). They are publicly available under accession PRJEB54772. Assembly of the *Dyadobacter* sp. HH091 genome is available *via* IMG/MER^3^ using the IMG ID 2842103827.

## Results

### Symbiont *Dyadobacter* sp. HH091 attached to the surface of *Micrasterias radians* MZCH 672

**Figure 1:**
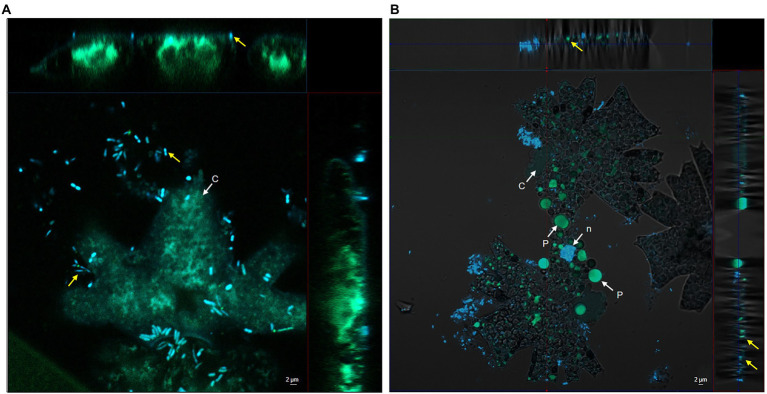
Confocal microscope including Z-Stack images of *Dyadobacter* sp. HH091 expressing eGFP (yellow arrows) found in a close proximity to *Micrasterias radians* MZCH 672. Autofluorescence Quenching Kit was used to lower the autofluorescence of chlorophyll of the microalga. Structures: c chloroplast, n nuclear region, p pyrenoid. Scale bar = 2 μm in each micrograph. **(A)** First day of incubation. **(B)** Third day of incubation.

Based on our previous research, we were intrigued to examine the bacterial colonisation of the microalga *M. radians* MZCH 672. CLSM was used to observe the interaction process between *Dyadobacter* sp. HH091 and *M. radians*. The co-culture of *M. radians* with *Dyadobacter* sp. expressing eGFP are shown in Figure 1. In addition, Z-Stack microscopy was employed to generate a more detailed and higher resolution image of the microalgal contact site with its symbiont. Our results showed, that symbiotic bacterial cells were found in close proximity of the alga after 1 day of incubation (Figure 1A). More nearby contacts were identified *via* CLSM between the host microalga and its symbiont on the third day of incubation (Figure 1B). At Figure 1A bacterial cells are found close to algal cells, while Figure 1B demonstrates the penetration of the symbiont into its host’s cell wall. These experiments revealed the presence of direct contacts between *M. radians* and symbiotic *Dyadobacter* sp. HH091 cells through their surrounding and tight interaction, promising the mutual exchange of various substances between the two partners.

We examined co-cultures of HH091 grown together with *M. radians* and compared its relative growth performance with the antibiotic-treated algal control cultures over a time period of 20 days (Supplementary Figure S1). To identify the difference in the growth of algal cultures (with and without HH091) we used the optical density measurement (Supplementary Figure S1). In these tests first hints of visible difference were observed after 3–4 days.

### RNA seq identifies active genes for host-symbiont interaction pathways

**Figure 2:**
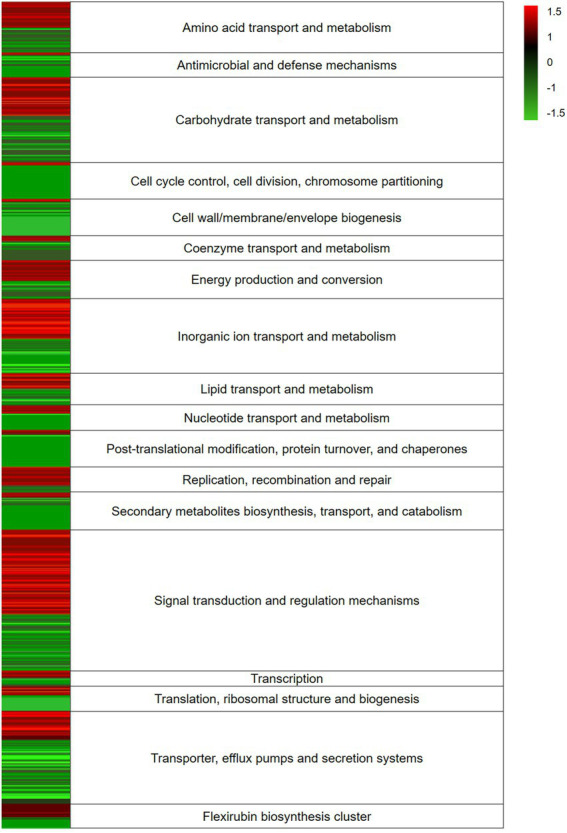
Heatmap of expression levels of differentially expressed genes (DEGs) response of *Dyadobacter* sp. HH091 in co-culture with *Micrasterias radians*. RNA-Seq analysis was performed using the Tuxedo strategy, the heatmap was generated using the Expression Import Service of the Pathosystems Resource Integration Center, PATRIC, the absolute value of log2 Ratio > 1.5. Color key: 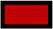 up-regulated genes, 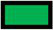 down-regulated genes.

Transcriptome analysis was applied to indicate highly active genes involved into bacteria-algal interaction. In total, we obtained 43 million (mio) reads of bacteria data after trimming. The data are the result of three replicates with each replicate producing between 4 and 8 mio reads (Supplementary Table S1). The RNAseq data covered a significant portion of the bacterial genome and the affiliated pathways. During data preprocessing low quality transcripts were filtered, resulting in 1,530 genes to be studied (Supplementary Table S2). RNA-Seq analysis was performed using the Tuxedo strategy, the heatmap (Figure 2) was generated using the Expression Import Service of the Pathosystems Resource Integration Center, PATRIC, the absolute value of log_2_ Ratio > 1.5 (Kim et al., 2013, 2015; McClure et al., 2013).

The expression levels of the DEGs response of *Dyadobacter* sp. HH091 in co-culture with *M. radians* are depicted in the heatmap (Figure 2). The heatmap reflects the expression of genes affiliated with overall mechanisms described in categories. The highest number of transcripts belongs to carbohydrate transport and metabolism, inorganic ion transport and metabolism, signal transduction and regulation mechanisms, and transporter, efflux pumps and secretion systems.

**Figure 3:**
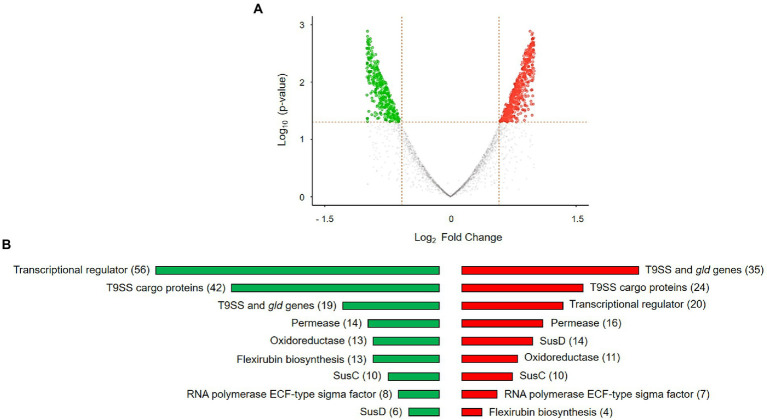
DEGs in *Dyadobacter* sp. HH091 co-cultured with *M. radians* MZCH 672 compared with control dataset. **(A)** Volcano plot is highlighting the DEGs in Dyadobacter sp. x-axis: log2, large-scale fold changes; y-axis: –log10 of the value of p showing the statistical significance. Each point corresponds to one gene. The points above the vertical and horizontal dotted lines represent log2FC ≥ 0.58 and value of *p* < 0.05. A volcano plot was generated using A Shiny app ggVolcanoR. **(B)** Function profile of differentially expressed genes (DEGs) in *Dyadobacter* sp. HH091 is presenting the groups of highly active genes. Total number of genes are shown in brackets. Color key: 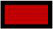 up-regulated genes, 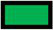 down-regulated genes.

The distribution of gene expression between *Dyadobacter* sp. HH091 co-cultured with *M. radians* and control samples is represented by the volcano plot (Figure 3A). The volcano plot was constructed to compare the two groups using ggVolcanoR. A total of 1,530 differentially expressed genes (DEGs) were identified from the dataset (Figure 3A). Among them, 612 and 918 genes were up-regulated and downregulated, respectively, between two groups according to their log_2_FC and *p*-values. Function profile of the DEGs in *Dyadobacter* sp. HH091 is shown in Figure 3B. The studying of the transcriptome of the strain HH091 co-cultured with its microalgal host unveiled the multifaceted combination of mechanisms required for and/or affiliated with T9SS, as well as T9SS cargo proteins, Sus proteins (SusC and SusD), TonB-dependent receptors, cAMP-binding proteins, oxidoreductases, aminotransferases, cytochrome c, numerous transcriptional regulators, including LuxR solos, and flexirubin biosynthesis. The highest number of up-regulated genes belongs to T9SS cargo proteins (42), transcriptional regulators (56), Sus proteins (SusC (10) and SusD (6)), permeases (14), and oxidoreductases (13). Most down-regulated genes are related to oxidoreductases (11), T9SS cargo proteins (24), SusC (10) and SusD proteins (14), T9SS components and Gld proteins (35), permeases (16), and transcriptional regulators (20). Intriguingly, flexirubin biosynthesis mechanism involved 13 up-regulated and 4 down-regulated genes.

### Transcriptome analysis indicated highly active genes of T9SS mechanism and flexirubin biosynthesis cluster

**Figure 4:**
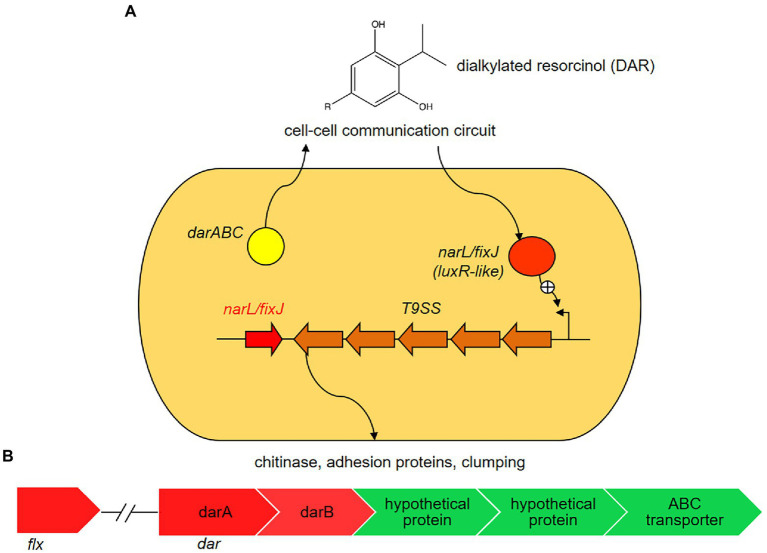
Proposed model of flexirubin or dialkylresorcinol (DARs) biosynthesis in *Dyadobacter* sp. HH091. In the proposed model, *Dyadobacter* sp. HH091 communicates *via* DARs and represents a novel quorum sensing (QS) circuit (Brameyer et al., 2015). It consists of the *dar* operon and a neighboring gene encoding a *luxR* solo (*narL/fixJ*). NarL/FixJ shares 46% identity and 47% similarity with the LuxR solo PluR of *P. luminescens* (IMG 2597490348), LuxR-type receptor serving for QS. The proposed QS circuit genes, adjacent to T9SS genes, genes affiliated with gliding motility and protein secretion, possibly upregulates several mechanisms, including T9SS, gliding motility and protein secretion. **(A)** Expression levels of DEGs involved into flexirubin biosynthesis: *dar* and *flx* clusters, and transport systems (ATP binding cassettes (ABC) and hypothetical proteins). Color key: 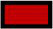 up-regulated genes, 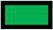 down-regulated genes. **(B)** Expression levels of differentially expressed genes (DEGs) involved into flexirubin biosynthesis: *dar* and *flx* clusters, and transport systems (ATP binding cassettes (ABC)-transporters and hypothetical proteins).

By a combination of comparative genome and transcriptome analyses we identified a cluster of genes presumably involved in flexirubin biosynthesis, which was performed using the STRING database (Szklarczyk et al., 2021). This cluster includes two genes, *darA* and *darB*, with likely roles in flexirubin synthesis, and other genes that could be involved in localization of flexirubin pigments (Supplementary Table S3). The flexirubin biosynthesis cluster of *Dyadobacter* sp. HH091 consists of the *dar* operon and a neighboring gene encoding LuxR solo (NarL/FixJ). NarL/FixJ shares 46% identity and 47% similarity with the LuxR solo PluR of *Photorhabdus luminescens* (Brameyer et al., 2015). In *P. luminescens* PluR performs as a LuxR-type receptor serving for QS. Based on these observations we proposed the model of flexirubin/dialkylresorcinol (DAR) biosynthesis in HH091, which consists of QS circuit genes possibly up-regulating several mechanisms like T9SS, gliding motility and protein secretion (Figure 4). These QS circuit genes are found to be adjacent to T9SS genes, genes affiliated with gliding motility and protein secretion (genes coding for gliding motility-associated-like proteins, T9SS type A sorting domain-containing proteins, chitin binding proteins, peptidoglycan-associated proteins, and PorT family protein).

Additional studying of homologs showed the presence of these genes in the representative genomes of the phylum Bacteriodota *Flavobacterium johnsoniae*, *Flavobacterium psychrophilum* (McBride et al., 2009) and *Chitinophaga pinensis* (Schöner et al., 2014), and among the members of the phylum Proteobacteriota *Photorhabdus asymbiotica* (Brameyer et al., 2015) and *Pseudomonas aurantiaca* (Nowak-Thompson et al., 2003). Responsible for flexirubin biosynthesis, genes *darA* and *darB* are similar to *F. johnsoniae*, which were previously identified to be engaged in biosynthesis of 2-hexyl-5-propyl-alkylresorcinol (McBride et al., 2009). In addition to *darA* and *darB*, other genes in this cluster are predicted to encode enzymes involved in lipid synthesis and some of these enzymes likely have roles in flexirubin synthesis (Supplementary Table S3). This cluster includes numerous genes, such as acyl carrier protein, (3-oxoacyl)-acyl carrier protein synthase, acyl-CoA thioester hydrolase, histidine ammonia-lyase, 1-acyl-sn-glycerol-3-phosphate acyltransferase, beta-ketoacyl synthases, and beta-hydroxyacyl-(acyl carrier protein) dehydratase, including several ABC-2-type transporters known to be entangled in the localization of flexirubin (McBride et al., 2009).

Experimental identification and validation of flexirubin confirmed its production by *Dyadobacter* sp. HH091 (Supplementary Figure S2). Cells were photographed before treatment (I), after exposure to 50 μl of 10 M KOH (II), and after exposure to KOH followed by exposure to 42 μl 12 M HCl (III). Flexirubin-positive cells were yellow at neutral pH (I and III) and orange/red under alkaline conditions (II).

### Proposed model of T9SS in *Dyadobacter* sp.

Highly active genes within this transcriptome belong to T9SS mechanism and gliding motility (Supplementary Table S4). Overall, 18 genes (*gldA*, *gldB*, *gldD*, *gldF*, *gldG*, *gldH*, *gldI*, *gldJ*, *gldK*, *gldL*, *gldM*, *gldN*, *sprA*, *sprE*, *sprF*, *sprT*, *porU* and *porV*), required for gliding motility and protein secretion, and/or involved in T9SS (Hunnicutt and Mcbride, 2000; McBride and Braun, 2004; Braun et al., 2005; Lauber et al., 2018; McBride, 2019; Hennell James et al., 2021; Trivedi et al. 2022; Veith et al., 2022), were identified among DEGs (Supplementary Table S2).

Besides that, a high number of transcripts was observed among genes responsible for polysaccharides utilization. That can also elucidate the up-regulation of genes coding for T9SS, while in commensal and environmental bacteroidotal species the T9SS is characteristically used to secrete enzymes that enable the organisms to utilize complex polysaccharides as a carbon source (Veith et al., 2013; Hennell et al., 2021).

Among up-regulated genes we identified different GHs and cell surface glycan-binding lipoproteins, known to be involved into plant and algal cell wall degradation mechanisms (Giovannoni et al., 2020). That included cellulose-degrading endoglucanases, hemicellulose-degrading xylosidases, pectin degradation proteins, starch-degrading enzymes, β-glucuronyl hydrolases, SusC and SusD family cell surface glycan-binding lipoproteins (Supplementary Table S2).

**Figure 5:**
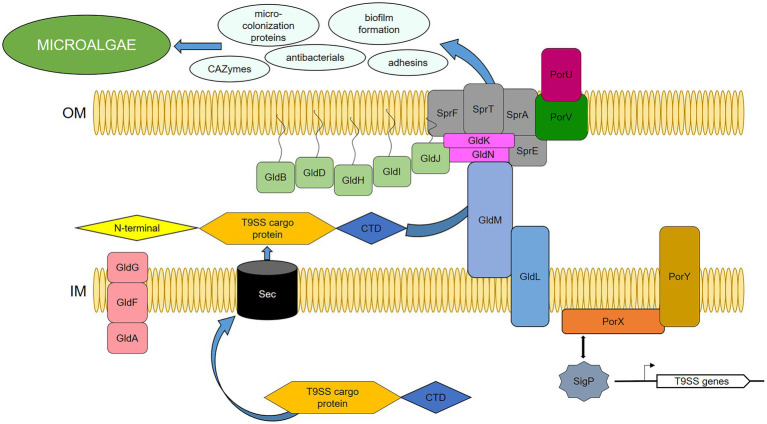
Proposed model of T9SS in *Dyadobacter* sp. HH091, serving as the secretion system of cargo-proteins. PorXY-SigP signalling system upregulates several components: T9SS category (GldK, GldL, GldM, GldN, SprA, SprE, SprF, SprT, PorU, PorV), and further Gld proteins (GldA, GldB, GldD, GldF, GldG, GldH, GldI, GldJ). C-terminal domain (CTD), N-terminal signal peptide (N-terminal), outer membrane (OM), inner membrane (IM).

Being concentrated on the components of T9SS, we identified highly active genes by transcriptome analysis of the strain HH091 co-cultured with its microalgal host. Domain guided annotation is based on conserved domains detected by STRING analysis of *Dyadobacter* sp. HH091 primary sequences against the genome of *Flavobacterium* spp. (Supplementary Table S4). Based on this analysis and previous researches (McBride and Zhu, 2013; Veith et al., 2013; Astafyeva et al., 2022), we proposed a model of T9SS including gliding motility proteins in *Dyadobacter* sp. HH091 (Figure 5). Intriguingly, genes, transcribing for the Gld motor proteins, were mostly down-regulated (*gldKLMN*), while genes coding for gliding motility-associated ABC transporter ATP-binding proteins were up-regulated. The transcriptome analysis suggests an explanation for this finding, because the symbiont possibly uses the T9SS not only for gliding motility, but also for the secretion of other proteins. Recent results by McBride and Saiki showed that nonmotile bacteroidotal members, such as *P. gingivalis*, *B. fragilis*, *B. thetaiotaomicron*, *B. vulgatus*, *P. distasonis*, and *Salinibacter ruber*, have homologs of genes, that have functions essential for protein secretion, but not for motility (Saiki and Konishi, 2007; McBride et al., 2009). Figure 5 represents a model of the T9SS including proteins required for gliding motility and/or protein secretion of *Dyadobacter* sp. HH091. This model includes the T9SS category (GldK, GldL, GldM, GldN, SprA, SprE, SprF, SprT, PorU, PorV), multiple PorXY-SigP signalling system components, and further Gld proteins (GldA, GldB, GldD, GldF, GldG, GldH, GldI, GldJ).

Most of the *gld* and T9SS genes are found to be adjacent to genes coding for proteins, involved into biosynthesis of glycosyltransferases, cell surface proteins, lipoprotein export proteins, as well as antibacterials, adhesion factors, microcolonization development, and EPS production. Interestingly, the up-regulated adjacent genes are also affiliated with cargo proteins of the T9SS. T9SS cargoes possess a conserved C-terminal domain (CTD) and an N-terminal signal peptide, and carry a CTD as a secretion signal, which is cleaved and replaced with anionic lipopolysaccharide by transpeptidation for extracellular anchorage to the outer membrane (OM) (Kulkarni et al., 2017; Mizgalska et al., 2021, 22; Gorasia et al., 2022). In this research, DEGs covered 42 up-regulated and 24 down-regulated genes affiliated with T9SS cargo proteins (Supplementary Table S5).

Along this detailed dataset investigation, the high activity of genes related to secretion systems and other entangled mechanisms underline the ability of *Dyadobacter* to perform the interaction with microalga and enable its dominance in many diverse environments.

## Discussion

The most comprehensive and fundamental understanding of microbial metabolic pathways in a multispecies system, as well as symbiotic and competitive interactions, is required to provide scientific and theoretical bases for the interaction mechanisms between microalgae and other microorganisms. The presented results promote not only the development of effective methods for simultaneous cultivation of algae, they also encourage the increasing the efficiency of microalgal biomass growth and associated production of valuable compounds.

### Flexirubin biosynthesis conceivably involved into microalgae-bacteria interaction

Our transcriptome analysis of *Dyadobacter* sp. HH091 co-cultured with microalga *M. radians* revealed highly active genes affiliated with the cluster of flexirubin biosynthesis. This cluster includes *darA* and *darB genes*, homologs of *F. johnsoniae* UW101 (McBride et al., 2009) and *C. pinensis* (Schöner et al., 2014).

Flexirubin is a pigment consisting of a ω-(4-hydroxyphenyl)-polyene carboxylic acid chromophore, esterified with a 2,5-dialkylresorcinol (DAR), also known as novel and widespread bacterial signalling molecule (Nowak-Thompson et al., 2003; Abt et al., 2011; Schöner et al., 2014). Genes coding for the biosynthesis of these pigments are found in many bacteroidotal genomes, including *Flavobacterium psychrophilum, Flavobacterium johnsoniae* (McBride et al., 2009), *Leadbetterella byssophila (Abt et al.,*^ 2011^*), Chryseobacterium artocarpi* (Venil et al., 2016), *Chryseobacterium rhizoplanae* sp. nov. (Kämpfer et al., 2015), *Flavobacterium maris* sp. nov. (Romanenko et al., 2015), and *Flavobacterium tilapiae* sp. nov. (Chen et al., 2013). Homologs of *darA*, a dialkylresorcinol condensing enzyme, and *darB*, a 3-oxoacyl-[acyl-carrier-protein] synthase III protein, were previously identified using bioinformatics tools within the genome analysis of our model organism *Dyadobacter* sp. HH091 (Astafyeva et al., 2022).

Another interesting point, is that on the plant-bacteria interaction model, flexirubin also performs as free radical scavenging antioxidant protecting from the attack of free radicals (Combes and Finet, 1997; Schöner et al., 2015). The antioxidant potential *via* hydrogen donating ability of flexirubin has been shown through the assessment using different assays such as radical scavenging activities, lipid peroxide inhibition and ferrous chelating ability (Mogadem et al., 2021). Several studies show that microalgae produce reactive oxygen species (ROS) to get an advantage in the competition for resources against other algae, be a way to prevent fouling bacteria, and act as a signalling mechanism between cells (Marshall et al., 2005). Furthermore, ROS, such as superoxide (O_2_^−^), hydrogen peroxide (H_2_O_2_), and hydroxyl radical (•OH), are thought to be produced as antibacterial agents and involved in oxidation or reduction of necessary or toxic metals (Palenik et al., 1987). Former investigation of microalga *Micrasterias* spp., demonstrated that ROS are constantly generated as by-products of general metabolic cellular pathways and can be over-produced in response to stress (Darehshouri and Lütz-Meindl, 2010; Lütz-Meindl, 2016; Felhofer et al., 2021). Our results indicate, that *Dyadobacter* sp. HH091 uses flexirubin hybrid pigments to protect itself from ROS produced by microalga, which explains this interaction, making it possible for microalgal symbiont to have a tight contact with its host.

### T9SS tangled in the symbiotic interactions of *Dyadobacter* with microalgae

The presence of different secretion systems suggests that *Dyadobacter* sp. HH091 and microalgae possess a signal exchange system allowing establishment and maintenance of a symbiosis that includes adhesion factors, microcolonization development, EPS production, and biofilm formation factors, which are important for the institution of a successful symbiosis. Previously, a comprehensive set of cell surface-associated proteins required for host cell invasion was described for other bacterial model organisms (Foster et al., 2014; Kusch and Engelmann, 2014; Hecker et al., 2018). All of these mechanisms express particular cocktails of factors that facilitate niche adaptation that include cell-host attachment, microcolonization and biofilm formation. Genes coding for the cell surface-associated proteins and secretion systems are mainly up-regulated in *Dyadobacter* sp. HH091, expecting them to be crucial for the microcolonization process because they establish interaction with the host. Cell-host interaction and adhesion factors, as well as microcolonization development, and biofilm formation succeed to a closely interaction and an exchange of growth-promoting substances between the symbiont and microalga.

Surface exposed proteins that are covalently or non-covalently bound to the cell surface and proteins are secreted into the extracellular matrix using different secretion mechanisms (Dreisbach et al., 2010; Ythier et al., 2012; Solis et al., 2014; Hecker et al., 2018). Secreted proteins accommodate the majority of virulence factors, enzymes required for nutrient acquisition or cell spreading, immune evasion proteins that can bypass the immune system or interfere with components of the complement system and many others. Overall, secretion systems are known to transport effector proteins into the cytosol of eukaryotic cells that allows the direct communication and modification of the host cells, additionally suppressing any activity of competitive microorganisms (Wooldridge, 2009). *Dyadobacter* sp. HH091 has many unique features together with the complex of different secretion systems, which are available to arbitrate secretion of proteins across the outer membrane, including T9SS, a complex translocon found only in some species of the Bacteroidota phylum (Lasica et al., 2017; Astafyeva et al., 2022).

A complex translocon of T9SS, including *gld* and *spr* genes, and *porXY-sigP* signalling system components, are proposed to serve as the secretion system of cargo-proteins. The T9SS cargo proteins have a conserved C-terminal domain (CTD) that enables them pass *via* T9SS and an N-terminal signal peptide that guides T9SS cargo proteins through the Sec system (Veith et al., 2013; Kulkarni et al., 2017). The CTD signal has been identified to be of two types, type A and type B (Kulkarni et al., 2017; Gorasia et al., 2020). Subsequent to the early *Dyadobacter* genome studies (Astafyeva et al., 2022), high activity of T9SS cargo proteins has been observed at this transcriptome analysis as well. It resulted in 48 up-regulated and 24 down-regulated genes, affiliated with T9SS cargo proteins of both types (Supplementary Table S5).

*gldA, gldF* and *gldG* encode components of an ATP-binding cassette (ABC) transporter that is required for motility and/or for the protein secretion (Agarwal et al., 1997; Hunnicutt et al., 2002). Genes encoding lipoproteins required for gliding (*gldB*, *gldD*, *gldH*, *gldI*, and *gldJ*) have also been identified (Hunnicutt and Mcbride, 2000; Hunnicutt and McBride, 2001; McBride and Braun, 2004; Braun and McBride, 2005). GldK, GldL, GldM, and GldN are each required for efficient motility and chitin utilization, indicating that Gld proteins may function in both gliding and chitin utilization (Braun et al., 2005). SprA is required for secretion of SprB and RemA and utilization of chitin (Nelson et al., 2007). In *F. johnsoniae*, SprA has been identified as the major translocon protein of T9SS, and it is hypothesized that SprA of *Dyadobacter* sp. HH091 can also have the same function (Lauber et al., 2018). Down-regulated gene coding for SprF is known to be essential for the secretion of SprB to the cell surface, but is not required for the secretion of extracellular chitinase (Rhodes et al., 2011). That also gives a hint that the symbiont possibly utilizes T9SS for the secretion of other proteins and not only involved in gliding motility.

### Polysaccharide utilization is a crucial aspect of microalgae-bacteria interaction

T9SS is known to be tangled in the secretion of polysaccharide utilization proteins (Braun et al., 2005; Kharade and McBride, 2014). Previously, it was shown that the major chitinase (ChiA) in *F. johnsoniae* is fully secreted from the cell in soluble form by T9SS and is essential for chitin degradation (McBride and Zhu, 2013; Kharade and McBride, 2014; Larsbrink et al., 2016).

Based on genome and transcriptome analyses, presumably, *Dyadobacter* sp. HH091 has a complex of carbohydrate utilization domains for digestion of microalgae cell wall hemicelluloses, such as cellulose, xylan or mannan fibrils, and extensive matrix polysaccharides. Numerous carbohydrate-active enzymes predicted to encode GHs and esterases that could be involved in the degradation of microalgal cell wall hemicelluloses were highly active within transcriptome datasets (Supplementary Table S2). In addition, candidates like xylanases, β-xylosidases, arabinofuranosidases, and beta-glucuronidases involved in xylan digestion, β-mannosidases involved in mannan digestion, and candidate β-glycosidases and endoglucanase that could be involved in xyloglucan digestion were also identified.

Data obtained from transcriptome analysis allows to better understand the nature of the involvement of bacterial polysaccharide utilization genes into bacteria-algae liaison. In our previous study, we observed that the genome of given symbiont possesses a wide assortment of CAZymes predicted to breach algal cell wall (Astafyeva et al., 2022). Deep investigation of transcriptome datasets unveiled the presence of these genes among DEGs. We observed that a significant number of genes (82) identified belonging to functions vital for carbohydrate transport and metabolism, including different GHs families, which are known to be involved into plant polysaccharides degradation (Kumar et al., 2017). For example, many up-regulated transcripts are affiliated with genes responsible for biosynthesis of GH5, GH13, GH25, GH30 and GH43 families enzymes, which function as effectors with roles in the degradation of plant polysaccharides (Rovenich et al., 2016; Snelders et al., 2018). These enzymes are known for acting as cellulose-degrading (Chang et al., 2016), starch-degrading (DeBoy et al., 2008), and catalysing hemicellulose and removing xyloses from xyloglucan (Glass et al., 2013; Bradley et al., 2022). Additionally, it was uncovered that genes affiliated with the synthesis of GH88 CAZyme, utilizing polysaccharide lyase activity to degrade pectins (Cantarel et al., 2009), was also up-regulated. Another highly active genes, coding for xylose isomerases, belong to CAZyme family GH43 that generally display specificity for arabinose-containing substrates. These gene combination reflects the competence of the symbiont to utilize starch and the complex of arabinan side-chains of pectin-rich cell walls as important nutrients (Ha et al., 2005; DeBoy et al., 2008).

Overall, our transcriptome analysis clearly showed, that bacteria can profit through the degradation of algal polysaccharides, while microalgae are being supplied with the repertoire of growth-promoting substances. The results of this research will serve as an efficient tool in further investigations of symbiotic microalgal–bacteria interactions. The remarkable benefit of a co-cultivation of microalgae and bacteria will have commercial and environmental positive impacts into the microalgal cultivation in the future.

## Data availability statement

The datasets presented in this study can be found in online repositories. The names of the repository/repositories and accession number(s) can be found at: Genbank, ON237360.

## Author contributions

YA and IK contributed to experimental design, lab work of metatranscriptomic, bioinformatics, and physiological analytical approaches, and writing of the research article. YA contributed to lab work of metatranscriptomic approaches and to assembly of metatranscriptomic datasets and bioinformatics approaches. MG and YA contributed to lab work of microscopic and analytical approaches. WS and IK contributed to general experimental design and writing of the research article. All authors contributed to the article and approved the submitted version.

## Funding

This work was in part supported by the DAAD, and the BMBF programs MarBioTech (FKZ 031A565) and AquaHealth (FKZ 031B0945C).

## Conflict of interest

The authors declare that the research was conducted in the absence of any commercial or financial relationships that could be construed as a potential conflict of interest.

## Publisher’s note

All claims expressed in this article are solely those of the authors and do not necessarily represent those of their affiliated organizations, or those of the publisher, the editors and the reviewers. Any product that may be evaluated in this article, or claim that may be made by its manufacturer, is not guaranteed or endorsed by the publisher.

## Footnotes

^1^ www.patricbrc.org

^2^ www.cazy.org

^3^ https://img.jgi.doe.gov

## Supplementary material

SUPPLEMENTARY FIGURE S1 Growth measurement (OD 750nm) of *Micrasterias radians* MZCH 672 in co-culture with the strain *Dyadobacter* sp. HH091. Increased growth rate (OD 750nm) can be observed in the co-culture with HH091 compared to the antibiotic-treated *M. radians* culture.

SUPPLEMENTARY FIGURE S2 Identification and validation of flexirubin pigments. Analysis of *Dyadobacter* sp. HH091, Maribacter dokdonemsis (yellow-pigment control, no flexirubin identified, Yoon et al., 2005), and Escherichia coli DH5α (negative control) strains for the presence of flexirubin pigments. Cells were photographed before treatment (I), after exposure to 50 µL of 10 M KOH (II), and after exposure to KOH followed by exposure to 42µL 12 M HCl (III). Flexirubin-positive cells were yellow at neutral pH (I and III) and orange/red under alkaline conditions (II).

SUPPLEMENTARY TABLE S2 Differentially expressed genes (DEGs) of transcriptome dataset of *Dyadobacter* sp. HH091 co-cultured with *M. radians*.

SUPPLEMENTARY TABLE S5 Differently expressed genes (DEGs) coding for the T9SS cargo proteins.
