# Supplementary figures and images for: Interplay between the microalgae Micrasterias radians and its symbiont Dyadobacter sp. HH091
Source: Front Microbiol. 2022 Oct 13;13:1006609. doi: 10.3389/fmicb.2022.1006609 (PMC9606717; doi:10.3389/fmicb.2022.1006609)

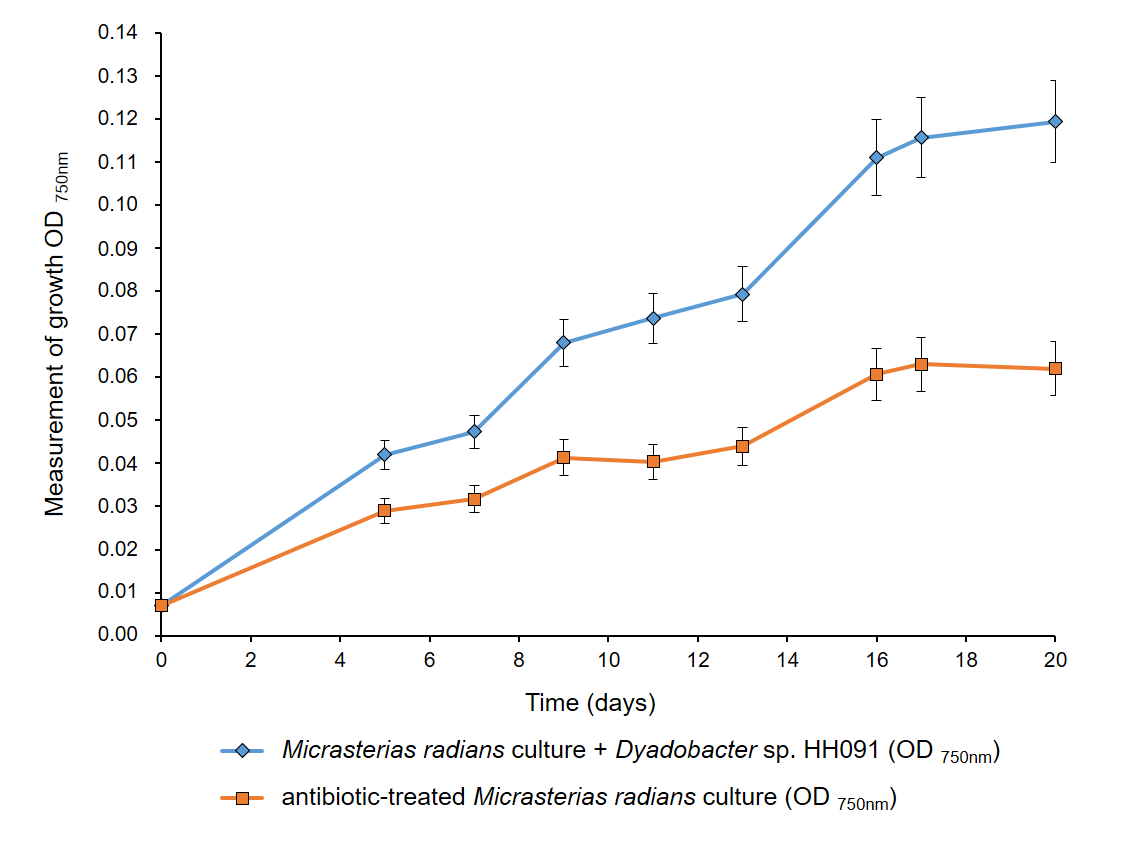

Supplement: SUPPLEMENTARY FIGURE S1 — Growth measurement (OD 750nm) of Micrasterias radians MZCH 672 in co-culture with the strain Dyadobacter sp. HH091. Increased growth rate (OD 750nm) can be observed in the co-culture with HH091 compared to the antibiotic-treated M. radians culture. [file Image_1.TIF]

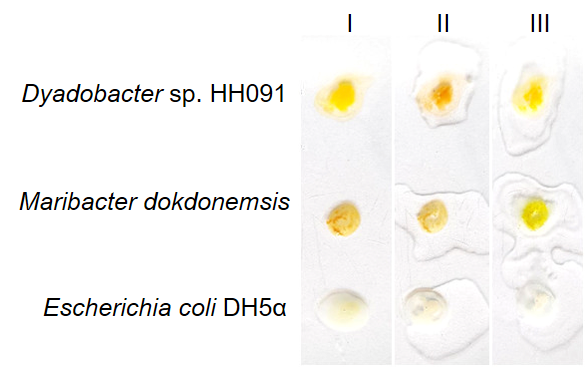

Supplement: SUPPLEMENTARY FIGURE S2 — Identification and validation of flexirubin pigments. Analysis of Dyadobacter sp. HH091, Maribacter dokdonemsis (yellow-pigment control, no flexirubin identified, Yoon et al., 2005), and Escherichia coli DH5α (negative control) strains for the presence of flexirubin pigments. Cells were photographed before treatment (I), after exposure to 50 µL of 10 M KOH (II), and after exposure to KOH followed by exposure to 42µL 12 M HCl (III). Flexirubin-positive cells were yellow at neutral pH (I and III) and orange/red under alkaline conditions (II). [file Image_2.TIF]
