# Supplementary material for: Interplay between the microalgae Micrasterias radians and its symbiont Dyadobacter sp. HH091
Source: Front Microbiol. 2022 Oct 13;13:1006609. doi: 10.3389/fmicb.2022.1006609 (PMC9606717; doi:10.3389/fmicb.2022.1006609)
Supplement: Supplementary file 5 [file Table_1.DOCX]

**Supplemental TABLE S1:** Overall numbers of sequences generated for the transcriptome datasets.

|  | |  |
| --- | --- | --- |
| **Reads Illumina (filtered)** | |  |
| Total no. |  | 43,164,382 |
| Average length (bp) |  | 151 |
| Duplicates (%) |  | 65.8 |
| GC (%) |  | 46 |
