# Supplementary material for: Interplay between the microalgae Micrasterias radians and its symbiont Dyadobacter sp. HH091
Source: Front Microbiol. 2022 Oct 13;13:1006609. doi: 10.3389/fmicb.2022.1006609 (PMC9606717; doi:10.3389/fmicb.2022.1006609)
Supplement: Supplementary file 6 [file Table_3.DOCX]

**TABLE S3:** Predicted gene clusters for flexirubin biosynthesis in *Dyadobacter* sp. HH091. Domain guided annotation is based on conserved domains detected by STRING analysis of *Dyadobacter* sp. HH091 primary sequences against the genome of *Flavobacterium* spp (IMG 2511231122, IMG 644736369).

| Gene ID | Gene Product Name | Pfam/Families | Gene | Organism | Identity (%) | AA Length |
| --- | --- | --- | --- | --- | --- | --- |
| 2842105285 | 3-hydroxyacyl-[acyl-carrier-protein] dehydratase | pfam07977-FabA; COG0764===3-hydroxymyristoyl/3-hydroxydecanoyl-(acyl carrier protein) dehydratase | FCOL_11760, FabA/FabZ | *Flavobacterium columnare* (strain ATCC 49512 / CIP 103533 / TG 44/87) | 36 | 119 |
| 2842105287 | peptidoglycan/xylan/chitin deacetylase (PgdA/CDA1 family), flxP | [pfam01522](http://pfam.xfam.org/family/PF01522)- Polysacc_deac_1; [COG0726](http://www.ncbi.nlm.nih.gov/Structure/cdd/cddsrv.cgi?uid=COG0726) - Peptidoglycan/xylan/chitin deacetylase, PgdA/CDA1 family | Fjoh_1086, flxP | *Flavobacterium johnsoniae* UW101 | 31 | 256 |
| 2842105288 | hypothetical protein | [pfam13723](http://pfam.xfam.org/family/PF13723)– Ketoacyl-synt_2 | Fjoh_1087 | *Flavobacterium johnsoniae* UW101 | 31 | 321 |
| 2842105289 | 3-oxoacyl-[acyl-carrier-protein] synthase-1 | pfam02801===Ketoacyl-synt_C<<>>pfam00109===ketoacyl-synt; COG0304===3-oxoacyl-(acyl-carrier-protein) synthase | Fjoh_1088 | *Flavobacterium johnsoniae* UW101 | 52 | 393 |
| 2842105291 | acyl carrier protein | pfam00550===PP-binding; COG0236===Acyl carrier protein | Fjoh_1089 | *Flavobacterium johnsoniae* UW101 | 57 | 85 |
| 2842105292 | hypothetical protein |  | Fjoh_1090 | *Flavobacterium johnsoniae* UW101 | 31 | 204 |
| 2842105293 | 3-oxoacyl-[acyl-carrier-protein] synthase-1 | pfam00109===ketoacyl-synt<<>>pfam02801===Ketoacyl-synt_C; COG0304===3-oxoacyl-(acyl-carrier-protein) synthase | Fjoh_1093 | *Flavobacterium johnsoniae* UW101 | 39 | 358 |
| 2842105294 | acyl-CoA thioester hydrolase | pfam13279===4HBT_2; COG0824===Acyl-CoA thioesterase FadM | Fjoh_1094 | *Flavobacterium johnsoniae* UW101 | 40 | 139 |
| 2842105295 | predicted hotdog family 3-hydroxylacyl-ACP dehydratase | COG4706===Predicted 3-hydroxylacyl-ACP dehydratase, HotDog domain | Fjoh_1101 | *Flavobacterium johnsoniae* UW101 | 28 | 138 |
| 2842105296 | predicted LPLAT superfamily acyltransferase | pfam03279===Lip_A_acyltrans; COG4261===Predicted acyltransferase, LPLAT superfamily | Fjoh_1104 | *Flavobacterium johnsoniae* UW101 | 39 | 288 |
| 2842105297 | acyl carrier protein | pfam00550===PP-binding; COG0236===Acyl carrier protein | Fjoh_1105 | *Flavobacterium johnsoniae* UW101 | 47 | 88 |
| 2842105299 | methionine-R-sulfoxide reductase | pfam01641===SelR; COG0229===Peptide methionine sulfoxide reductase MsrB | Fjoh_0270 | *Flavobacterium johnsoniae* UW101 | 36 | 141 |
| 2842105302 | 3-oxoacyl-[acyl-carrier-protein] synthase-1 | pfam00109===ketoacyl-synt<<>>pfam02801===Ketoacyl-synt_C; COG0304===3-oxoacyl-(acyl-carrier-protein) synthase | Fjoh_1106 | *Flavobacterium johnsoniae* UW101 | 64 | 406 |
| 2842105303 | 3-oxoacyl-[acyl-carrier protein] reductase (short-chain dehydrogenase); flxB | pfam13561===adh_short_C2; COG1028===NAD(P)-dependent dehydrogenase, short-chain alcohol dehydrogenase family | Fjoh_1107, flxB | *Flavobacterium johnsoniae* UW101 | 65 | 243 |
| 2842105304 | histidine ammonia-lyase; flxA | pfam00221===Lyase_aromatic; COG2986===Histidine ammonia-lyase | Fjoh_1109, flxA | *Flavobacterium johnsoniae* UW101 | 52 | 509 |
| 2842105305 | 1-acyl-sn-glycerol-3-phosphate acyltransferase; flxU | pfam03176===MMPL<<>>pfam13847===Methyltransf_31<<>>pfam01553===Acyltransferase; COG0204===1-acyl-sn-glycerol-3-phosphate acyltransferase<<>>COG4258===Predicted exporter | Fjoh_1078, flxU | *Flavobacterium johnsoniae* UW101 | 28 | 1277 |
| 2842105306 | all-trans-retinol 13,14-reductase; flxV | pfam13450===NAD_binding_8; COG1233===Phytoene dehydrogenase-related protein | Fjoh_1077, flxV | *Flavobacterium johnsoniae* UW101 | 44 | 509 |
| 2842105307 | flavin-dependent dehydrogenase; flxK | pfam01494===FAD_binding_3; COG0644===Dehydrogenase (flavoprotein) | Fjoh_1110, flxK | *Flavobacterium johnsoniae* UW101 | 47 | 410 |
| 2842105308 | 3-oxoacyl-(acyl-carrier-protein) synthase | pfam13723===Ketoacyl-synt_2; COG0304===3-oxoacyl-(acyl-carrier-protein) synthase | Fjoh_1087 | *Flavobacterium johnsoniae* UW101 | 34 | 355 |
| 2842105340 | 3-oxoacyl-(acyl-carrier-protein) synthase | pfam02801===Ketoacyl-synt_C<<>>pfam00109===ketoacyl-synt; COG0304===3-oxoacyl-(acyl-carrier-protein) synthase | Fjoh_1088 | *Flavobacterium johnsoniae* UW101 | 45 | 399 |
| 2842105341 | acyl carrier protein | pfam00550===PP-binding; COG0236===Acyl carrier protein | Fjoh_1089 | *Flavobacterium johnsoniae* UW101 | 48 | 88 |
| 2842105342 | ABC-2 type transport system permease protein; darJ | pfam12698===ABC2_membrane_3; COG0842===ABC-type multidrug transport system, permease component | Fjoh_1095, darJ | *Flavobacterium johnsoniae* UW101 | 32 | 425 |
| 2842105343 | ABC-2 type transport system ATP-binding protein; darI | pfam00005===ABC_tran; COG1131===ABC-type multidrug transport system, ATPase component | Fjoh_1096, darI | *Flavobacterium johnsoniae* UW101 | 52 | 249 |
| 2842105344 | hypothetical protein (BtrH); darH | pfam16169===DUF4872<<>>pfam14399===BtrH_N | Fjoh_1097, darH | *Flavobacterium johnsoniae* UW101 | 48 | 343 |
| 2842105345 | hypothetical protein |  | Fjoh_1098 | *Flavobacterium johnsoniae* UW101 | 53 | 132 |
| 2842105346 | 3-oxoacyl-[acyl-carrier-protein] synthase-3; darB | pfam08541===ACP_syn_III_C; COG0332===3-oxoacyl-[acyl-carrier-protein] synthase III | darB | *Flavobacterium johnsoniae* UW101 | 50 | 380 |
| 2842105347 | hypothetical protein; darA |  | darA | *Flavobacterium johnsoniae* UW101 | 39 | 303 |
| 2842108407 | UDP-3-O-[3-hydroxymyristoyl] N-acetylglucosamine deacetylase/3-hydroxyacyl-[acyl-carrier-protein] dehydratase; FabZ | pfam03331 - LpxC  pfam07977 – FabA; COG0774 - UDP-3-O-acyl-N-acetylglucosamine deacetylase  COG0764 - 3-hydroxymyristoyl/3-hydroxydecanoyl-(acyl carrier protein) dehydratase | fabZ | *Flavobacterium johnsoniae* UW101 |  |  |
