# Supplementary material for: Interplay between the microalgae Micrasterias radians and its symbiont Dyadobacter sp. HH091
Source: Front Microbiol. 2022 Oct 13;13:1006609. doi: 10.3389/fmicb.2022.1006609 (PMC9606717; doi:10.3389/fmicb.2022.1006609)
Supplement: Supplementary file 7 [file Table_4.DOCX]

**TABLE S4:** Predicted components of the type IX protein secretion system (T9SS) in *Dyadobacter* sp. HH091. Domain guided annotation is based on conserved domains detected by STRING analysis of *Dyadobacter* sp. HH091 primary sequences against the genome of *Flavobacterium* spp (IMG 644736369, IMG 640753027, IMG 2731957834).

| Gene ID | Gene Product Name | Pfam/Families | Gene | Organism | Identity (%) | AA Length |
| --- | --- | --- | --- | --- | --- | --- |
| 2842106854 | GldA; Gliding motility-associated ABC transporter ATP-binding protein | TIGR03522; pfam00005 - ABC_tran | Fjoh_1516, GldA | *Flavobacterium johnsoniae* UW101 | 62 | 235 |
| 2842108806 | GldB; gliding motility-associated protein | TIGR03514 | Fjoh_1793, GldB | *Flavobacterium johnsoniae* UW101 | 27 | 347 |
| 2842104678 | GldC; gliding motility-associated protein | TIGR03515; pfam19937 - GldC-like | Fjoh_1794, GldC | *Flavobacterium johnsoniae* UW101 | 42 | 113 |
| 2842109028 | GldD; gliding motility-associated protein | TIGR03512 | Fjoh_1540, GldD | *Flavobacterium johnsoniae* UW101 | 36 | 198 |
| 2842106522 | GldE; gliding motility-associated protein | TIGR03520; pfam00571 – CBS; pfam03471 - CorC_HlyC | Fjoh_1539, GldE | *Flavobacterium johnsoniae* UW101 | 36 | 443 |
| 2842106486 | GldF; Gliding motility-associated ABC transporter permease protein | pfam12679 - ABC2_membrane_2 | Fjoh_2722, GldF | *Flavobacterium johnsoniae* UW101 | 49 | 242 |
| 2842109240 | GldG; gliding-associated putative ABC transporter substrate-binding component | TIGR03521; pfam09822 - ABC_transp_aux | Fjoh_2721, GldG | *Flavobacterium johnsoniae* UW101 | 35 | 552 |
| 2842110280 | GldH; gliding motility-associated lipoprotein | TIGR03511; pfam14109 - GldH_lipo | Fjoh_0890, GldH | *Flavobacterium johnsoniae* UW101 | 25 | 161 |
| 2842108793 | GldI; gliding motility-associated protein | pfam00254 - FKBP_C | Fjoh_2369, GldI | *Flavobacterium johnsoniae* UW101 | 24 | 256 |
| 2842109190 | GldJ; gliding motility-associated protein | TIGR03530; pfam03781 - FGE-sulfatase | Fjoh_1557, GldJ | *Flavobacterium johnsoniae* UW101 | 42 | 417 |
| 2842108285 | GldK; gliding motility-associated lipoprotein | TIGR03529; pfam03781 - FGE-sulfatase | Fjoh_1853, GldK | *Flavobacterium johnsoniae* UW101 | 35.2 | 357 |
| 2842108286 | GldL; gliding motility-associated protein | TIGR03513 | Fjoh_1854, GldL | *Flavobacterium johnsoniae* UW101 | 35.2 | 270 |
| 2842108287 | GldM; gliding motility-associated protein | TIGR03517; pfam12080 - GldM_C;  pfam12081 - GldM_N | Fjoh_1855; GldM | *Flavobacterium johnsoniae* UW101 | 23 | 530 |
| 2842108288 | GldN; gliding motility associated protein | TIGR03523; pfam19841 – GldN | Fjoh_1856; GldN | *Flavobacterium johnsoniae* UW101 | 41 | 342 |
| 2842109196 | type IX secretion system PorP/SprF family membrane protein | TIGR03519; pfam11751 - PorP_SprF | FP0017; PorP/SprF | *Flavobacterium psychrophilum*  (strain ATCC 49511 / DSM 21280 / CIP 103535 / JIP02/86) | 23 | 342 |
| 2842110175 | type IX secretion system PorP/SprF family membrane protein | TIGR03519; pfam11751 - PorP_SprF | FP0017; PorP/SprF | *Flavobacterium psychrophilum* (strain ATCC 49511 / DSM 21280 / CIP 103535 / JIP02/86) | 19 | 336 |
| 2842108613 | SprB | TIGR04131; pfam13585 - CHU_C | FP0016, sprB | *Flavobacterium psychrophilum* (strain ATCC 49511 / DSM 21280 / CIP 103535 / JIP02/86) | 35 | 683 |
| 2842109195 | SprB | TIGR04131; pfam13585 - CHU_C;  pfam18911 - PKD_4 | FP0016, sprB | *Flavobacterium psychrophilum* (strain ATCC 49511 / DSM 21280 / CIP 103535 / JIP02/86) | 34 | 961 |
| 2842106852 | RemA | TIGR04183; pfam19081; Ig-like domain CHU_C associated; pfam18962 Secretion system C-terminal sorting domain | RCH33_2786; remA | *Flavobacterium daejeonense* RCH33 |  | 1369 |
| 2842105604 | SprE; tetratricopeptide (TPR) repeat protein | COG0457 - Tetratricopeptide (TPR) repeat; pfam13174 - TPR_6 | Fjoh_1051; sprE | *Flavobacterium johnsoniae* UW101 | 23 | 726 |
| 2842106491 | SprA; cell surface protein | TIGR04189; pfam14349 - SprA_N | FP2121, sprA | *Flavobacterium psychrophilum* (strain ATCC 49511 / DSM 21280 / CIP 103535 / JIP02/86) | 32 | 2408 |
| 2842106773 | PorU | pfam01364 - Peptidase_C25 | FP1388, porU | *Flavobacterium psychrophilum* (strain ATCC 49511 / DSM 21280 / CIP 103535 / JIP02/86) | 26 | 1129 |
| 2842106772 | PorV | pfam19572 - PorV | Fjoh_1555; porV | *Flavobacterium johnsoniae* UW101 | 33 | 385 |
